# 

**Published:** 1883-10

**Authors:** 


PAGE

ORIGINAL COMMUNICATIONS.—The United States Goverment in its relations to Contagious Animal Diseases and the Veterinary Profession. Frank S. Billings, V.S. ........ 263
Contraction of the Foot in the Horse. Thomas Walley, M.R.C.V.S. ........ 273
Physical Diagnosis. E. Benjamin Ramsdell, M.D. ........ 275
Castrations Statistics. W. S. Adams, V.S ........ 281
Comparative Physiology of Menstruation. Alfred Wiltshire, M.D. F.R.C.P. ........ 284
Cutaneous Disease in the Horse similar to Tinea Fovosa in Man. Profsssor R. Bassi ........ 295
Lameness. E. A. MacLellan, D.V.S ........ 298
EDITORIAL.—Glanders—U. S. Veterinary Profession and the R.C.V S—New York Cattle Quarantine—Innoculation for Pleuro-Pueumonia—Sander's Mission—How the Gastric Juice of the Horse's Stomach is Secreted—The Tubercle-Bacillus in Hens—State Veterinary Associations—The New Harvard Veterinary School ........ 306–117
CASE DEPARTMENT.—Extensive Cystic Disease of the Liver in a Pig—Fracture of the Os Suffraginus with complete recovery—Hepatitis in a Dog—Cases Reported by Wm. Herbert Lowe, D.V.S. ........ 313–317
OBITUARY ........ 317
REVIEWS.—A Text Book of the Practice of Equine Medicine—Books and Pamphlets Received ........ 317–819
PROGRESS OF VETERINARY SCIENCE.—A New Parasiticide—Cattle Liable to Abort—Itch in the Cat—Amputation of Penis—Chicken Cholera—Fraeture of Femur in Puma—Pneumothorax in a Coati—Acute Farcy in Man—Sarcoma in a Common Fowl—The Principal Disease of the Hock—Treatment of Poll Evil—Treatment of Quitters—Cancer of the Stomach—Thickening of the Villous Membrane of the Stomach ........ 320–325
NEWS AND MISCELLANY.—Personal—Trichnosis from Eating Horseflesh—Elephant Poisoned by Prussic Acid—Victims to Wolves—Orang-Otang in London—Blinkers—Clover Bloat—Shoeing a Kicking Mule or Horse—Removal of Dead Animals—Diseased Meat in Glasgow—The Boston Dog Destroyer—What shall he do with them—The Cattle Plague—Domestication of the Horse—Maternal Impression—Death from Hydrophobia, Fatal Result of the scratch of a Cat—Blood Poisoning in the Lower Vertebrate—Bequests to the Dick Veterinary College and the University of Edinburgh—Moor Tops for Stable Bedding—City Horse Railways—Rubbing the Tail—The Diseases of Monkeys—Remarkable Case of Cataract in a Dog—The Stereoscope in the Study of Anatomy ........ 326–332

